# Contributing barriers to loss to follow up from antenatal care services in villages around Addis Ababa: a qualitative study

**DOI:** 10.1186/s12905-021-01290-9

**Published:** 2021-04-07

**Authors:** Zergu Tafesse Tsegaye, Hailemariam Segni Abawollo, Binyam Fekadu Desta, Tsega Teferi Mamo, Atrie Fekadu Heyi, Mestawot Getachew Mesele, Addisu Dabesa Lose

**Affiliations:** JSI/ USAID Transform: Primary Health Care Activity, Addis Ababa, Ethiopia

**Keywords:** ANC, Loss to follow up from ANC, ANC defaulter, ANC dropout, USAID transform:Primary health care

## Abstract

**Background:**

Problems during pregnancy, childbirth and postpartum are the major contributors to maternal and perinatal morbidity and mortality. Focused antenatal care is an intervention set to provide basic services for pregnant women, to reduce morbidity and mortality related to pregnancy. In Ethiopia, there is a significant loss to follow up from antenatal care services between the first and fourth visits. The aim of this study is to explore the potential contributing barriers to loss to follow up of pregnant women from antenatal care services in villages around Addis Ababa, the capital city of the country.

**Methods:**

A qualitative research method was used, where 20 in-depth interviews (zonal, woreda and health center managers, midwives and health extension workers were the participants) and three focus group discussions (mothers who were lost to follow up, mothers who completed four sessions of antenatal care visits, and community volunteers were the participants) were conducted. A qualitative data analysis software, ATLAS.ti 8, was applied.

**Results:**

Inability to deliver essential antenatal care services which occurs primarily due to shortage of the required medical equipment, drugs, and other supplies is a major barrier for sustainable attendance of antenatal care services followed by poor care, respect, and receptiveness of service providers. Lack and cost of transport as well as partners’ approval and support were also claimed to be part of the major barriers. Community culture and pervious maternal experiences as well as maternal sociodemographic factors like maternal age at time of pregnancy and educational status of mothers were also reported as barriers to seeking and completion of antenatal care services.

**Conclusions:**

Both demand and supply side barriers play a significant role in the loss to follow up from antenatal care services. Availing essential antenatal care services, closer to the community by improving the infrastructure, health workforce and supply chain system is recommended.

**Supplementary Information:**

The online version contains supplementary material available at 10.1186/s12905-021-01290-9.

## Background

Globally, every year, 303,000 women die from preventable causes related to pregnancy and childbirth. Additionally, 2.6 million stillbirths and 2.7 million newborn deaths occur annually. Ninety three percent of these deaths occur in low and lower-middle-income countries. Antenatal care (ANC) is crucial for the prevention of maternal and newborn deaths and stillbirths. Currently in the world, 86% of pregnant women access at least one ANC with skilled providers during pregnancy and 78% deliver with the assistance of skilled birth attendants [1].

The maternal mortality ratio of Ethiopia is 401/100,000 live births with 14,000 annual maternal deaths, almost all of which are preventable [2]. The most common causes of maternal death in the country are hemorrhage, preeclampsia/eclampsia, sepsis/infection, and prolonged/obstructed labor [3]. The ANC-1 coverage in the country is 74% and ANC-4 + is 43% showing a huge gap (31%) between ANCs one and four [4].

The standard of services rendered in the country during ANC visits was also found to be low as evidenced by low early ANC initiation (20%), [5] and low coverage of essential ANC services like blood pressure measurement, urine and blood tests (55.8%), [1] and iron supplementation (42%) [6].

The proportion of health facilities in the country that possess blood pressure measurement apparatus is 59%; the capacity for syphilis testing is 42%, 27% for HIV testing, 20% for hemoglobin determination, 33% for blood glucose determination, and 46–47% for urine testing; iron supplements and tetanus toxoid vaccines are available in 61% and 65% of facilities, respectively [6].

In the Debremarkos town of north-eastern Ethiopia, the proportion of dropouts from the maternity continuum of care was found to be 32.2%. The major contributing variables associated with the dropouts were having no exposure to media, unplanned pregnancies, and having < 4 ANC follow up [7].

In the country, especially in Oromia region including Finfinne special zone, recent administrative reports showed a significant loss to follow up between ANC-1 to ANC-4 with paucity of evidence on contributing barriers for the loss to follow up. Hence, it is worth looking into the major gaps in ANC to devise possible cost-effective and high impact interventions that improve the loss to follow up from ANC services.

The aim of this study is to explore the potential contributing barriers for loss to follow up from ANC services and solutions to mitigate those barriers. To specifically know both the demand and supply side barriers it is better to directly hear the problem from health managers, service providers, community volunteers, and mothers. Hence, qualitative study design was used.

## Methods

### Design

A qualitative study design was employed.

### Setting and period

The study was conducted in villages around Addis Ababa, the capital city of Ethiopia. These villages are part of the Finfinne Special Zone in Oromia region. Finfinne Special Zone was selected purposely due to resource issues as it is located close to the capital city. The study was conducted in July 2020.

### Study participants

**Fig. 1:**
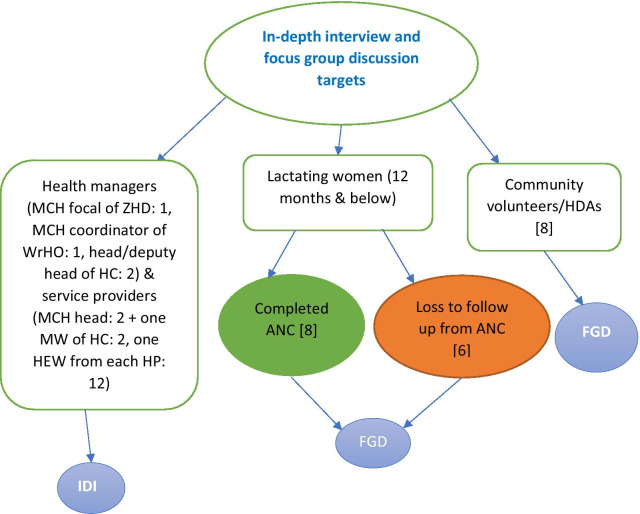
Targets for IDIs and FGDs

The study targeted health managers, health workers and health extension workers (HEWs) for the in-depth interviews (IDIs) and community volunteers/health development armies (HDAs) and women who had given birth recently (in the past 12 months) and were lost to follow up from ANC and those who attended at least four ANC visits for the focus group discussions (FGDs) (Fig. 1). All the women who participated in the study were above 18 years of age.

### Sample size and sampling method

The zonal health department, one woreda in the zone, two health centers in the selected woreda, and all health posts within catchment of those selected two health centers were purposefully selected based on their high rates of loss to follow up from ANC based on administrative reports of the health centers. Twenty IDIs were conducted where the participants were a maternal and child health (MCH) focal person in the zonal health department (ZHD), a MCH coordinator of the woreda health office (WrHO), head/deputy heads + MCH heads + a midwife per the two health centers, and one HEW from each of the health posts (HP). Three FGDs were conducted consisting of six to eight participants per FGD. The three FGD groups were mothers who gave birth within the past 12 months and had at least four ANC visits during the index pregnancy (eight mothers), mothers who gave birth within the past 12 months and were lost to follow up from ANC during the index pregnancy (six mothers), and community volunteers/HDAs (eight HDAs). The final sample sizes of both IDIs and FGDs were also determined based on level of saturation of the information required.

### Data collection

An IDI guide was developed and administered to health managers at zone, woreda and service providers at health center and health post levels. The IDI guide contains an information sheet, an informed consent form and interview guide points which include if health facilities are providing the standard ANC services, if they have the necessary materials for the purpose, barriers for low ANC uptake and loss to follow up, and how to mitigate those barriers. Two different FGD guides were developed and one was used to facilitate the FGDs of the two categories of women and the other guide to facilitate FGD of the HDAs. Both the FGD guides contain an information sheet, an informed consent form and discussion guide points which include services rendered during ANC visits of the recent pregnancy, way of treatment by health workers, support from relatives, motivators for completing ANC visits, barriers for loss to follow up, areas of improvement at different levels for better ANC service delivery and prevention of loss to follow up from ANC for the mothers and if health facilities are providing the standard ANC services, if they have the necessary materials for the purpose, barriers for low ANC uptake and loss to follow up, and how to mitigate those barriers for the HDAs. Both the IDI and FGD guides are found as Additional file 1.

Trained data collectors who are Master of Public Health graduates with relevant health backgrounds conducted both the IDIs and FGDs. Two people facilitated each of the IDIs and FGDs. Voice recorders were used during both the IDIs and FGDs and both were conducted using the local language, Afan Oromo. After completion of interviews and discussions, each of the records and other relevant documents were put in a secure and safe place.

### Transcription and translation of IDIs and FGDs

Transcription and translation of the IDIs and FGDs were conducted by a consultant who has ample experience in transcription and translation of IDIs and FGDs and is fluent in the local language used to conduct the IDIs and FGDs.

### Data analysis

Analysis of the study was conducted using thematic analysis through a qualitative data analysis software. ATLAS.ti 8 software was used to code and categorize the transcription. The first transcripts were used to frame the coding structures. Two research team members independently coded all transcripts then met and agreed on the coding structures and discussed the emerging themes. The identified contributing barriers for defaulting from ANC are summarized under two themes, demand and supply side barriers. Demand side barriers are individual, household or community characteristics that influence the demand for ANC services. Supply side barriers are those characteristics of the health system that exist beyond the control of potential health system users, including but not limited to health facilities, equipment, drugs, finances, and health workers.

### Ethical consideration

Ethical clearance was granted from the John Snow Incorporated (JSI) institutional review board (IRB), IRB REFERENCE: IRB # 20-16 E, and from Oromia regional health bureau. IDI and FGD participants have read information sheets and signed informed consent. Confidentiality was respected accordingly. We also confirm that all methods were carried out in accordance with relevant guidelines and regulations.

## Results

The IDI participants were female and male professionals working at different levels of the health system having different professional backgrounds and wide range of age. The FGD participants were only females in reproductive age with varying educational background and economic statuses. The identified contributing barriers for loss to follow up from ANC are summarized under two themes, demand and supply side barriers.

### Demand side barriers

Demand side barriers are presented under three major categories: sociodemographic and obstetric, individual-family-community, and access and geography.

#### Sociodemographic and obstetric barriers

Both IDI and FGD participants iterated some sociodemographic barriers affecting ANC attendance. Of these factors**,** those with unwanted pregnancies and older age mothers were not willing to attend ANC services expressing that they feel ashamed. One FGD participant said,

“Unwanted pregnancy is one of the barriers to attend ANC because mothers don’t want to expose their secrets to neighbors and health professionals. Hence, they want to stay at home”.

Maternal education was also mentioned several times as a barrier by the participants in both FGDs and IDIs. Participants of FGDs and IDIs stated that the educational status of mothers is one of the barriers in either facilitating or hampering the utilization of ANC services. An FGD participant mother stated,

“Inability to read the card is one of the challenges I faced many times. I tried to remember the appointment as much as possible and asked my children to read it for me. I think this is a problem of many other mothers too”.

Additionally, preference of specific sex of service providers and perceived problems related to the use of technology were reported as barriers. One attendant of an IDI reported,

“Since almost all midwives are females, pregnant women are comfortable using the services, but they are not comfortable with ambulance services as service providers are male”.

Moreover, one participant of an IDI reported,

“Some of the mothers attending ANC services have issues related to the use of technologies like ultrasounds, thinking that they will face problems in using them”.

#### Individual, family and community barriers

Participants of IDIs said that community level traditions and cultures are influencing practices of pregnant women significantly. A participant said,

“According to some cultures, pregnant women deliver at their parents’ place, and are forced to move there at third trimester and this could lead to defaulting from ANC”.

Lack of the decision-making capacity of pregnant women to attend health services by themselves is another barrier because they lack the power to decide on the spending of family resources. As ANC service attendance requires transport costs at a minimum, for women unable to make decisions on resources, distance from their health facility was mentioned as one of the barriers to attending ANC. In addition to the costs involved, clients in this assessment also frequently claimed that shortage of transportation to-and-fro facilities was one of the potential barriers for attending ANC services. A mother who participated in one of the FGDs said,

“For me, distance may be one reason. It is difficult for pregnant mothers to attend ANC services due to distance and lack of transport. It would be better if ANC services were given at health posts to minimize this problem”.

Community and pregnant women’s perception of the benefits of ANC services was also one of the barriers mentioned. A participant of an IDI said,

“The community discourages pregnant women by informing them there is no additional benefit from visiting a health center for ANC services”.

Individual and community experiences also influence personal decisions to utilize health services including ANC attendance. A participant of an IDI said,

“Peer pressure from people who didn’t get quality services during their visit influences others not to go for ANC services”.

In addition to that, previous maternal experiences regarding health facility attendance also influence women’s current practices. A participant reported,

“The effect of previous experience also matters; if a mother gave birth to a child without any follow up, she might think that there is no need for ANC services”.

In addition, some of the participants mentioned that if a mother did not experience any problems during her past pregnancy/pregnancies, she is likely to be reluctant to attend ANC services for subsequent pregnancies.

Personal problems like sickness or death of relatives and responsibilities that involve taking care of family members were also mentioned as barriers.

Poor family support, particularly poor partner support, was mentioned as one of the barriers. Some IDI participants reported that,

“Pressure from partner is one of the major barriers. Husbands may not be happy when their wives visit health centers for ANC services and some husbands are not willing to accompany their wives for some essential services like ANC”.

In addition, maternal preference of specific health facilities to attend for ANC and preterm labor were mentioned as reasons for not completing the minimum number of ANC visits.

Mothers in the rural parts of the country were overburdened with household chores and lack time to attend ANC services. One health facility staff reported,

“Mothers prefer to take care of their children at home than come for ANC services”.

In addition, women want to get fast services whenever they visit health facilities for different services. This is sometimes not possible and becomes source of disappointment. One FGD participant reported,

“There are complaints on waiting times to get services because health professionals often come late”.

Some of the health managers enrolled in IDIs informed that some mothers may not know that they are pregnant and that this may be the reason for late initiation of ANC.

#### Access and geographic barriers

According to some of the participants, the topography between residential areas of mothers and health facilities is a major challenge to access health services. An IDI participant from a health center said,

“Some of the ANC service utilizers at our health center are from rural kebeles where they need to cross a river (Awash). This is an obstacle for mothers coming for the services, especially during rainy seasons,”.

Additionally, distance of health centers from residential areas was mentioned as a barrier. Most of the participants of IDIs indicated that lack of transport and cost of transport are the major factors affecting the attendance of ANC services. Most of the health managers indicated that,

“Lack or shortage of transportation services and not being able to afford transportation costs were reported by some mothers”.

Mothers who participated in FGDs reported,

“Lack of transportation is a big problem. To solve this problem ANC services and delivery care should be given at health posts in every kebele”.

### Supply side barriers

Supply side barriers are presented under four major categories related to: health workers; shortage of medical equipment, drugs and other supplies; infrastructure and linkage; and health system management and leadership.

#### Health workers related barriers

Shortage of the required number and type of service providers at all levels, lack of hospitality and receptiveness, timely service provision, absenteeism from work, and lateness of service providers were some of the barriers mentioned. These barriers were highly influencing the sustainable utilization of services in facilities. One woman who participated in FGD said,

“If health professionals want to enhance ANC, they should respect their clients and not only to help a woman finish an ANC service but also to motivate others to come and attend ANC”.

Another woman also reported,

“I was forced to interrupt ANC service utilization at the health center due to mistreatment from service providers”.

FGD participants have also reported that they wanted to receive services from the same provider during different sessions of ANC visits but there was frequent change of service providers during different ANC visit times which was a reason for their dissatisfaction and interruption of the service. A participant of an IDI said,

“Having different service providers to attend a single pregnant woman at her ANC visits is one of the potential reasons for dissatisfaction and interruption as pregnant women are not comfortable to tell their entire health history to different people and this is evident as many of them ask for the person who provided the service initially and will leave if s/he is not around”.

#### Medical equipment, drugs, and other supplies shortage related barriers

Shortage of the required medical equipment was reported as one of the major barriers to the provision of a full package of ANC services in health facilities. One of the attendants of an IDI reported,

“Sometimes there is a disruption of ANC services when equipment for prevention of mother to child transmission of HIV, hemoglobin, reagents for syphilis tests and hepatitis B and C are out of stock”.

As per the report of health managers who participated in IDIs, there are often shortages in equipment required to provide full package of ANC services in health facilities including blood pressure apparatus and weighing scales. One of the participants mentioned,

“Absence of a full package of ANC services at health facilities creates dissatisfaction and in turn becomes a reason for defaulting”.

Shortage of drugs is also mentioned by one participant, saying,

“There are problems with sustainable supply of iron or folic acid in health facilities and with reporting of the utilization of these drugs”.

Additionally, another FGD participant reported that there are shortages of ANC related drugs saying,

“There is scarcity of ANC related medications. They were prescribed for me and I couldn’t find it in the health center, so I used to buy them from a private pharmacy monthly”.

Proper planning and reporting are the bases of good quality services at each level of the health system, but this is not always practiced. Some participants said,

“The facility focuses on reporting with little attention on planning and how to sustainably supply materials needed for essential services”.

#### Health facility infrastructure and linkage related barriers

Some of the health managers reported absence and frequent interruption of electric power as a barrier for providing basic ANC laboratory services leading to repeated appointments and hence loss to follow up of mothers from ANC. Additionally, some of the participants said that arrangement of the ANC room and shortage of rooms in health posts are areas of discomfort for mothers to sustainably attend ANC services.

On some occasions, when HEWs refer mothers from the community to health centers, the referral health center does not provide the expected service and mothers get disappointed and interrupt the service. A HEW said,

“Although most services are supposed to be provided at the health center, because of lack of services there, we receive complaints from pregnant women we have referred”.

#### Health system management and leadership related barriers

HEWs reported that health centers do not send back laboratory test results of mothers referred from health posts for the tests making the delivery of ANC services during subsequent visits difficult which contributes to loss to follow up of mothers due to dissatisfaction on the service. During IDIs the HEWs said,

“Though we refer using a referral form, health centers are not sending the results of the tests they have carried out for pregnant women to our health post for follow up”.

Additionally, some HEWs reported,

“There is no support from health centers”.

Lack of a regular supportive supervision is mentioned as one of the factors affecting ANC services provision contributing to loss to follow up of mothers. Some IDI participants said,

“The lack of regular supportive supervision from zones and woredas to identify and fill gaps is also affecting the ANC services provided to mothers”.

Additionally, events such as vaccination days, campaigns, and emergency management activities which occupy many health workers were also reported as reasons for not providing ANC services. Some service providers think that ANC services should start after 16 weeks of gestation.

Staff assignment in health facilities is usually dependent on professional areas of specialty and training. But sometimes this is not practically applied because of rotations, turnover and duty assignments. Based on the observation of participants, health workers were not willing to consult each other, and this resulted in compromised quality of services. One participant of an IDI recommended,

“There needs be consultation among service providers to improve quality of ANC services”.

Mothers complained that the services given to them at health facilities were not to the level of their expectations. Some of the participants reported that they had received some services like weight and blood pressure measurement during their visit to the health facilities but a few of them reported that they did not receive such services.

Participants reported that health posts were understaffed, which leads to closures of the facility when they are away on duty, and pregnant women missing their ANC schedule becoming disappointed. As per the report of participants,

“Closing of health posts due to house to house visits and campaign by HEWs is common with no one available to provide the services for mothers and children at the health posts in their absence”.

Mothers were not coming to ANC service as per the schedule, but they used to come to health facilities during market days which causes health workers to be overloaded as many people visit health facilities for different services during these days. In addition, language barrier between mothers and health care workers is mentioned as one of the barriers.

## Discussion

The results of this assessment showed that there are demand and supply side barriers influencing attendance of ANC. Demand side barriers that affect the utilization of ANC services by pregnant women include sociodemographic and obstetric barriers such as age at which the women get pregnant, unwanted pregnancies; individual, family and community related barriers including workload on women, lack of partners’ support, no autonomy for women in decision making, and pervious individual and community experiences. Additionally, poor access to health facilities, particularly health centers, due to lack of all-weather roads, lack of transport services and cost of transport were mentioned as barriers influencing utilization. The results of a systemic review on factors influencing the use of prenatal care also indicated similar findings [8]. Another study conducted in Somali region of Ethiopia also indicated that socio-demographic, economic status, cultural believes, past experiences, level of awareness, attitude toward the service, challenges in accessing transportation and shortage of supplies were identified as major barriers for ANC service utilization [9].

We identified supply side barriers also for loss to follow up from ANC services. Some of these are health workers related barriers in which lack of the required number and type of service providers in health facilities results in an appointment for pregnant mothers for another day. On occasions where there are shortages of midwives in health facilities, the overload of tasks forces them to re-schedule visiting mothers-to-be for another day and this means some of the women may not come back to get an ANC service. Moreover, according to this assessment, health workers’ lack of respect and perceptiveness were also among the major barriers for the consistent utilization of ANC services. Additionally, health workers’ absenteeism and turnover were some of the issues raised during the IDIs and FGDs. Lack of adequate numbers of laboratory technicians in health facilities was also raised repeatedly as a barrier to get the required quality ANC service. A qualitative study conducted in the Somali regional state of Eastern Ethiopia and in the North West of Ethiopia reflected similar results [9, 10].

The findings of this assessment also indicated that shortage of the required medical equipment, drugs, and other supplies were other key barriers to delivery of ANC services and hence loss to follow up. It was reported that there are shortages of some essential drugs like iron and folic acid in health facilities which leads to the rescheduling of mothers for another day resulting in disappointment of clients and interruption of the service. Additionally, shortages of the required equipment such as blood pressure apparatus and weighing scales were found to be barriers to providing ANC services resulting in disappointment and loss to follow up from ANC services. The bases for the shortages were poor planning on both the parts of managerial and health facility level staff.

This assessment also showed that lack of basic amenities like electricity were a determinant to providing ANC services to clients. It has been claimed by most of the participants that health facilities experience shortages in electricity or have frequent power interruptions which is a key barrier for providing laboratory services leading to a referral or rescheduling of appointments. This leads to dissatisfaction of clients and interruption of service utilization. Additionally, per the national direction, HEWs must refer mothers to health centers to attend the first and the fourth ANC services as there are some services missing at the health post level. Despite this, the referral health facilities are sometimes not able to provide the required services which deters clients from attending the facilities and results in the interruption of the services. Clients also complained about the distance of health facilities and lack of access to and cost of transportation to reach the referral health facilities resulting in interruptions of the service. A similar finding was reported in a qualitative study from Afar regional state which showed that the barriers to health facilities included distance, lack of transportation, sociocultural factors, and disrespectful care [11]. A mixed designed study in Bahir Dar Zuria Woreda also indicated similar findings that the socio-culture of the community, attitudes, experience, and perception of the existing services and service provisions were also determinants of ANC drop out [12].

An efficient management and provision of support are key elements to providing quality health services. Based on this assessment, the support and linkage between facilities and the management was found to be weak, affecting the timely supply of the required essential supplies, and enhancement of knowledge and skills of service providers. A qualitative study conducted in Jimma zone of South West Ethiopia reflected that the linkage between midwives and HEWs were found to be poor because of resource limitations and poor infrastructure [13].

### Limitations

The findings of this assessment should be interpreted taking into consideration the limitations of the study. The fact that it is purely dependent on qualitative information collected from different levels of the health system and was not triangulated with any quantitative data from the health system is a major limitation of the study.

## Conclusion

The demand side barriers of ANC service utilization and loss to follow up in Finfinne area special zone, Oromia region are age, educational status, unwanted pregnancy, preference of sex of service providers, perceived problems on use of technology, work load on women, lack of partners’ support, community culture and traditions, perception of the benefits of ANC service, and availability and cost of transportation.

The supply side barriers are shortage of medical equipment, drugs and other supplies; lack of hospitality and receptiveness, timely service provision, absenteeism from work, and lateness; mothers want to receive services from same provider over the different sessions of ANC visits, lack of full package of ANC services, lack or interruption of electric power, shortage of rooms, arrangement of rooms, non-favorable working environments, poor linkage and technical support between the different levels of facilities, and poor consultation among service providers.

Based on the findings of the study, it is recommended that full package of ANC services be availed closer to the community through improving the infrastructure, equipment, supplies, drugs, and staffing of health posts. Intensify capacity enhancement activities with a focus on motivated, competent, and compassionate (MCC) health workforce and make MCC part of the periodic performance evaluation of service providers and monitoring activities including integrated supportive supervisions. Deploy the required number and type of service providers in health facilities. Strengthen the supply chain system and linkage of facilities with policy level structures so that they identify major gaps and act timely. Demand for ANC be created through informing the community on benefits of ANC.

## Supplementary Information

**Additional file 1**. IDI and FGD guides.

## Data Availability

The datasets during and/or analyzed during the current study are available from the corresponding author on reasonable request.

## Abbreviations

ANC: Antenatal care
FGD: Focus group discussion
HDA: Health development army
HEW: Health extension worker
IDI: In-depth interview
JSI: John snow incorporated
MCC: Motivated competent compassionate
MCH: Maternal and child health
USAID: United States Agency for International Development
