# Supplementary material for: Contributing barriers to loss to follow up from antenatal care services in villages around Addis Ababa: a qualitative study
Source: BMC Womens Health. 2021 Apr 7;21:140. doi: 10.1186/s12905-021-01290-9 (PMC8028793; doi:10.1186/s12905-021-01290-9)
Supplement: Supplementary file 1 — Additional file 1. IDI and FGD guides. [file 12905_2021_1290_MOESM1_ESM.docx]

**Contributing barriers to loss to follow up from antenatal care services in villages around Addis Ababa: a qualitative study**

Zergu Tafesse Tsegaye^1^, Hailemariam Segni Abawollo^*1^, Binyam Fekadu Desta^1^, Tsega Teferi Mamo^1^, Atrie Fekadu Heyi^1^, Mestawot Getachew Mesele^1^, Addisu Dabesa Lose^1^

^1^ JSI/ USAID Transform: Primary Health Care Activity, Addis Ababa, Ethiopia.

^*^Corresponding author.

**In depth interview guide**

1. How do you rate the ANC coverage of your catchment area?

***(Probe****: Increasing over the past couple of years? ANC-1? ANC-4? Defaulters?)*

1. Do you think health facilities in your catchment area are providing to the standard/quality ANC services?

*(****Probe****: Early ANC? Are essential services (BP, urine test, blood test, counselling, iron and folic acid supplements) being provided regularly for all mothers coming for the service?)*

1. Are the necessary things to provide to the standard/quality ANC services available in your catchment area/health facilities?

*(****Probe****: Adequate human resource? Adequate medical equipment? Adequate other materials and supplies? Management/leadership support?)*

1. What do you think are the barriers/reasons for low coverage of ANC/early ANC and high ANC defaulter rate in the country generally and your catchment area specifically?

*(****Probe:*** *With possible barriers identified from local researches like economic constraints, rural residence, mother’s lack of awareness, mother’s low educational status, mother’s absence of exposure to media, shortage of female health care provider/mothers prefer female health care providers, husband’s disapproval/lack of male engagement, lack of acceptance by the community, absence of full ANC package services at health facilities (secondary to shortage of manpower, medical equipment and other supplies), lack of perceived benefits from ANC service, unplanned pregnancy, older age group, high parity with many living children, absence of problems/illnesses during index pregnancy, delayed initiation of ANC, etc.) (5, 6, 7, 8)*

1. What can be done to mitigate those barriers/factors at different levels?

*(****Probe****: Ministry of Health level, Regional Health Bureau level, Zonal Health Department level, Woreda Health Office level, Health Facility level, Health Post level, community level, individual level)*

1. Do you have any further questions/concerns you want to discuss?

***Thank participant and close the interview session.***

**Focus group discussion guide for mothers**

1. Where did you attend for ANC follow up during the recent/last pregnancy?

*(****Probe****: Health post? Health center? Hospital? Private clinic? Where do you think it is appropriate for pregnant mothers to attend for their ANC services?)*

1. What was done for you and your unborn baby at the health facility during your visit for ANC follow up?

*(****Probe****: Blood pressure measurement? Counselling? Urine test? Blood test? Iron and folic acid supplements? For how long you took the IFA supplements?)*

1. How were you treated at the health facility during your ANC follow up visits?

*(****Probe****: Woman friendly care was given (companion of her choice could go in with her, was treated with respect and dignity), was informed and involved at every decision made)*

1. Did you have someone in your life to provide you with emotional support during the pregnancy?

*(****Probe****: Accompany to health facility during ANC, support with household chores, remind her with appointments and medications)*

1. If you have **attended at least four ANC visits** during the recent/last pregnancy, what were the motivating factors for you to adhere with the ANC recommendations?

(***Probe***: Presence of problem, wanting a healthy baby, wanting to be healthy during and after pregnancy, health facility was nearby, female providers are present, partner supports the attendance, happy with the services, treated with respect and dignity at the facility, etc.) (5, 6, 7, 8, )

1. If you have **not attended at least four ANC visits** during the recent/last pregnancy, what were the barriers/factors which hindered you from adhering to the ANC recommendations?

(***Probe***: Economic, distance from health facility, lack of awareness, prior pregnancies were uneventful, non-woman friendly care at health facilities, absence of partner engagement, generally not happy with the services, full package of ANC is not provided, unplanned pregnancy, no problem during the index pregnancy, etc.) (5, 6, 7, 8)

1. What things do you think need improvement for better continuation of ANC visits for quality services?

(***Probe***: Things to be done at different levels like individual, partner, facility, leadership, etc.)

1. Do you have any further questions/concerns you want to discuss?

***Thank participants and close the focus group discussion session.***

**Focus group discussion guide HDAs**

1. How do you rate the ANC attendance of your surrounding community?

***(Probe****: Increasing over the past couple of years? ANC-1? ANC-4? Defaulters?)*

1. Do you think health facilities in your locality are providing to the standard/quality ANC services?

*(****Probe****: Early ANC? Are essential services (BP, urine test, blood test, counselling, iron and folic acid supplements) being provided regularly for all mothers coming for the service?)*

1. Do you think the necessary things to provide to the standard/quality ANC services available in nearby health facilities?

*(****Probe****: Adequate human resource? Adequate medical equipment? Adequate other materials and supplies? Management/leadership support?)*

1. What do you think are the barriers/reasons for low coverage of ANC/early ANC and high ANC defaulter rate in your community?

*(****Probe:*** *With possible barriers identified from local researches like economic constraints, rural residence, mother’s lack of awareness, mother’s low educational status, mother’s absence of exposure to media, shortage of female health care provider/mothers prefer female health care providers, husband’s disapproval/lack of male engagement, lack of acceptance by the community, absence of full ANC package services at health facilities (secondary to shortage of manpower, medical equipment and other supplies), lack of perceived benefits from ANC service, unplanned pregnancy, older age group, high parity with many living children, absence of problems/illnesses during index pregnancy, delayed initiation of ANC, etc.)*

1. What can be done to mitigate those barriers/factors at different levels?

*(****Probe****: Ministry of Health level, Regional Health Bureau level, Zonal Health Department level, Woreda Health Office level, Health Facility level, Health Post level, community level, individual level)*

1. Do you have any further questions/concerns you want to discuss?

***Thank participants and close the focus group discussion session.***
